# miR-24 and miR-205 expression is dependent on HPV onco-protein expression in keratinocytes^☆^

**DOI:** 10.1016/j.virol.2013.10.014

**Published:** 2014-01-05

**Authors:** Declan J. McKenna, Daksha Patel, Dennis J. McCance

**Affiliations:** a Biomedical Sciences Research Institute, University of Ulster, Coleraine, Co. Derry BT52 1SA, UK; b Centre for Cancer Research and Cell Biology, School of Medicine, Dentistry and Biomedical Science, Queen's University Belfast, Belfast BT9 7BL, UK

**Keywords:** microRNA, miR-24, miR-205, HPV, Keratinocytes, Differentiation

## Abstract

A screen of microRNA (miRNA) expression following differentiation in human foreskin keratinocytes (HFKs) identified changes in several miRNAs, including miR-24 and miR-205. We investigated how expression of Human Papilloma Virus Type-16 (HPV16) onco-proteins E6 and E7 affected expression of miR-24 and miR-205 during proliferation and differentiation of HFKs. We show that the induction of both miR-24 and miR-205 observed during differentiation of HFKs is lost in HFKs expressing E6 and E7. We demonstrate that the effect on miR-205 is due to E7 activity, as miR-205 expression is dependent on pRb expression. Finally, we provide evidence that miR-24 effects in the cell may be due to targeting of cyclin dependent kinase inhibitor p27. In summary, these results indicate that expression of both miR-24 and miR-205 are impacted by E6 and/or E7 expression, which may be one mechanism by which HPV onco-proteins can disrupt the balance between proliferation and differentiation in keratinocytes.

## Highlights

• miR-24 and miR-205 are induced during keratinocyte differentiation.
• This induction is lost in keratinocytes expressing HPV onco-proteins E6 and E7.
• miR-205 is dependent upon pRb expression.
• miR-24 targets p27 in cycling keratinocytes.

## Introduction

Over the past decade, a growing body of evidence has shown that microRNAs (miRNAs) play a fundamental role in the development, function and maintenance of tissues and cells in various organisms. miRNAs are small, non-coding RNA molecules that can regulate protein expression at the post-transcriptional level by targeting mRNAs for degradation or translational repression (Erson and Petty, 2008). It is now known that many miRNAs are implicated in several disease states, including heart disease (Bauersach and Thum, 2007; Latronico et al., 2007), viral infection (Sullivan and Ganem, 2005) and many different cancers (Cowland et al., 2007; Visone and Croce, 2009), leading to increased interest in the biology and function of individual miRNAs in various cell processes.

In skin physiology, the importance of miRNAs in skin epithelial development is particularly emphasized when their expression is repressed by epidermal-specific deletion of Dicer in mouse models, which results in several defects, such as epidermal evagination and abnormal hair follicle development, although it is worth noting that epidermal differentiation is apparently unaffected (Andl et al., 2006; Yi et al., 2006). To date, several individual miRNAs have been identified as playing fundamental roles in keratinocytes, including microRNA-205 (miR-205) and microRNA-24 (miR-24).

miR-205 is now known to play a fundamental role in epithelial biogenesis and maintenance (Qin et al., 2013) and has been widely studied in a number of settings. It is believed to act as a tumour suppressor miRNA, since its expression is down-regulated in a number of different cancers, although more recent evidence has also shown it may also promote cell proliferation in certain circumstances (Qin et al., 2013). For example, in both primary human epidermal keratinocytes and corneal epithelial keratinocytes, it has been shown to promote migration by regulating the lipid phosphatase SHIP2 (Yu et al., 2008, 2010). In contrast, its tumour suppressor function in oral keratinocytes has be\en linked to induction of interleukin-24 levels (Kim et al., 2013). Like miR-205, miR-24 also seems to play contrasting roles depending on the setting, but it is generally found to be up-regulated in various cancers including oral squamous cell carcinoma (Lin et al., 2010) and is postulated to have an oncogenic function. In keratinocytes, it has been implicated in regulation of differentiation by control of actin adhesion and various cytockeletal modulators affecting migration (Amelio et al., 2012), as well as contributing to FGF-β-mediated regulation of epithelial-to-mesenchymal tranisiton (EMT) through its targeting of Net1A (Papadimitriou et al., 2012). Taken together, these keratinocyte studies demonstrate that both miR-205 and miR-24 play important roles in keratinocyte proliferation and differentiation, with abnormal expression of either likely to result in altered cell behaviour.

This becomes important in considering the effect of HPV-16 infection of epithelial cells, since the effect of the viral onco-proteins E6 and E7 activity is known to impact upon miRNA expression (Zheng and Wang, 2011) and could contribute to the initiation of the tumourigenic process. We have previously reported that miR-203 expression in human foreskin keratinocytes (HFKs) is mediated by E6 activity through degradation of p53 (McKenna et al., 2010), and we concluded that it was likely that other miRNAs were similarly affected. Therefore, in this report, we investigate how the expression of miR-24 and miR-205 is affected by expression of HPV onco-proteins in HFKs during proliferation and differentiation.

## Results and discussion

**Fig. 1:**
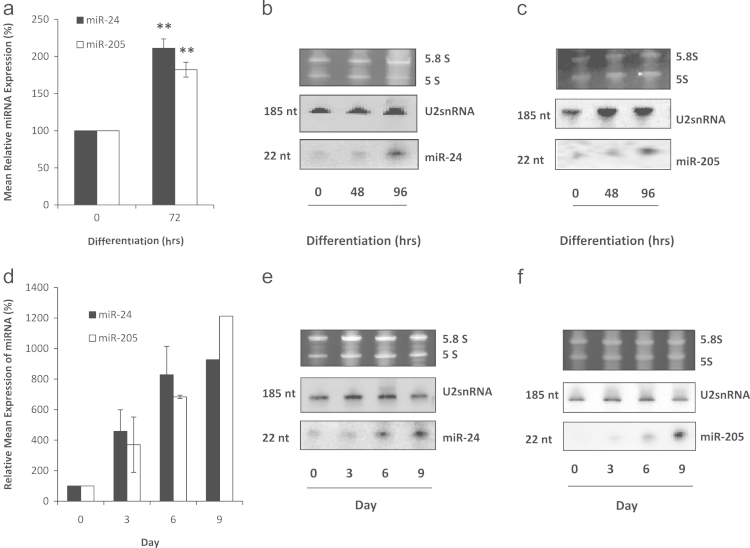
miR-24 and miR-205 are induced during keratinocyte differentiation. (a) RQ-PCR analysis of RNA samples harvested from HFKs following calcium-induced differentiation demonstrates that miR-24 and miR-205 expression increases significantly over time. These results were confirmed by Northern blotting analysis for miR-24 (b) and miR-205 (c). (d) RQ-PCR analysis of RNA samples harvested from organotypic rafts at different time points also demonstrate that miR-24 and miR-205 expression increases significantly over time. These results were confirmed by Northern blotting analysis for miR-24 (e) and miR-205 (f). For figures (a–c), all images and blots are representative of 3 independent experiments performed on separate batches of HFKs. Figures (d–f) are representative of two independent experiments performed on separate batches of HFKs. Data shown in graphs are mean±SE (Student *t*-test *p*-values: *p<0.05, **p<0.01, and ***p<0.001).

From an initial miRNA screen, carried out in collaboration with Eric Miska (University of Cambridge), we had noted the expression levels of several miRNAs in HFKs (Supplementary Table 1). Two highly expressed miRNAs were miR-205 and miR-24, both of which showed an increase in expression following differentiation, suggesting they were important in this process. We validated these screening results by measuring miR-24 and miR-205 expression in keratinocytes induced to differentiate by calcium treatment, and in organotypic rafts, which are 3-dimensional skin equivalents (McCance et al., 1988), derived from normal HFKs. Quantitative real-time polymerase chain reaction (RQ-PCR) (Fig. 1a) and Northern blotting (Fig. 1b and c) both show that miR-24 and miR-205 are significantly up-regulated during calcium-induced HFK differentiation. Likewise, increases in miR-24 and miR-204 were observed in the organotypic raft model of keratinocyte differentiation (Fig. 1d–f). Only two other studies have investigated these miRNAs in differentiation of keratinocytes and our results agree with their observations that miR-205 is up-regulated during differentiation of keratinocytes (Nissan et al., 2011), whilst miR-24 is up-regulated in murine keratinocytes during differentiation (Amelio et al., 2012). Indeed, both miRNAs have been associated with differentiation in different settings, suggesting they have a fundamental role in mediating the switch from proliferation to differentiation in different cell types.

**Fig. 2:**
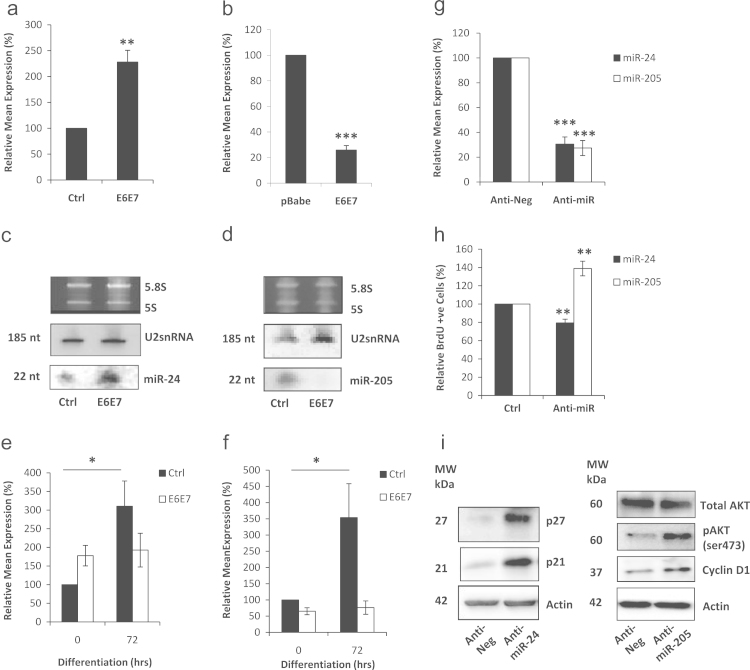
miR-24 and miR-205 expression correlates with proliferative capacity. (a) RQ-PCR analysis and (c) Northern blotting demonstrated that miR-24 expression was significantly increased in HFKs expressing E6 and E7. By contrast miR-205 was shown to be significantly decreased in the same cells by RQ-PCR (b) and Northern blotting (d). When these cells were induced to differentiate by calcium treatment, RQ-PCR shows that the up-regulation of (e) miR-24 expression and (f) miR-205 expression observed in control (Ctrl) HFKs is lost in HFKs expressing E6 and E7. (g) To test the effect of each miRNA on HFK proliferation, antagomiR inhibitors were separately used to knockdown levels of each miRNA, confirmed by RQ-PCR. (h) Proliferation, as measured by BrdU incorporation, is increased in HFKs in which miR-205 is inhibited and decreased in HFKs in which miR-24 is inhibited. (i) Western blots confirming that miR-24 knockdown results in induction of cell cycle inhibitory molecules, whilst miR-205 knockdown causes increased expression of cell cycle proliferation proteins. All images and blots are representative of three independent experiments performed on separate batches of HFKs. Data shown in graphs are mean±SE (Student *t*-test *p*-values: *p<0.05, **p<0.01, and ***p<0.001).

With this in mind, we wanted to examine the effect of E6 and E7 onco-proteins on these miRNAs, since studies by ourselves (McKenna et al., 2010) and others (reviewed in Zheng and Wang, 2011) have shown that cellular miRNA expression can be affected by HPV infection. We observed by RQ-PCR and Northern blotting that HFKs transduced to express both E6 and E7 onco-proteins resulted in increased miR-24 expression (Fig. 2a and c) and decreased miR-205 expression (Fig. 2b and d). Furthermore, we noted that the induction of both of these miRNAs during calcium-induced differentiation was lost in HFKs expressing E6 and E7 (Fig. 2e and f). This is a similar finding to that which we had previously observed for miR-203 (McKenna et al., 2010) and is further evidence that HPV infection of cells can disrupt miRNA expression.

Since miR-24 and miR-205 have putative roles as an oncogene and a tumour suppressor respectively, we wanted to specifically examine the effect of altering miR-24 and miR-205 expression on proliferation in cycling HFKs. Levels of both miRNAs were knocked-down separately in HFKs with specifically targeted antagomiR molecules (Fig. 2g). Knockdown of miR-205 resulted in significantly increased HFK proliferation, with ~40% more cells staining for BrdU incorporation than control cells, whilst knocking down of miR-24 significantly inhibited HFK proliferation by ~21% (Fig. 2h). As expected, when miR-24 was over-expressed in HFKs, we noted that HFK proliferation was significantly increased (data not shown). The proliferative effect of knocking down miR-205 was further illustrated by western blots showing increased activity of Akt pathway and increased Cyclin D1 levels (Fig. 2i). In the case of miR-24, we noted that knocking down expression resulted in an increase of p21 and p27, indicating an inhibition of the cell cycle (Fig. 2i). These results suggest that, in HFKs, miR-24 and miR-205 nominally behave in an oncogenic and tumour suppressor function respectively, observations which agree with the roles proposed for them by other studies in keratinocytes (Lin et al., 2010; Kim et al., 2013). With that in mind, we proceeded to look at each separately to investigate how these functions might be influenced in HFKs.

**Fig. 3:**
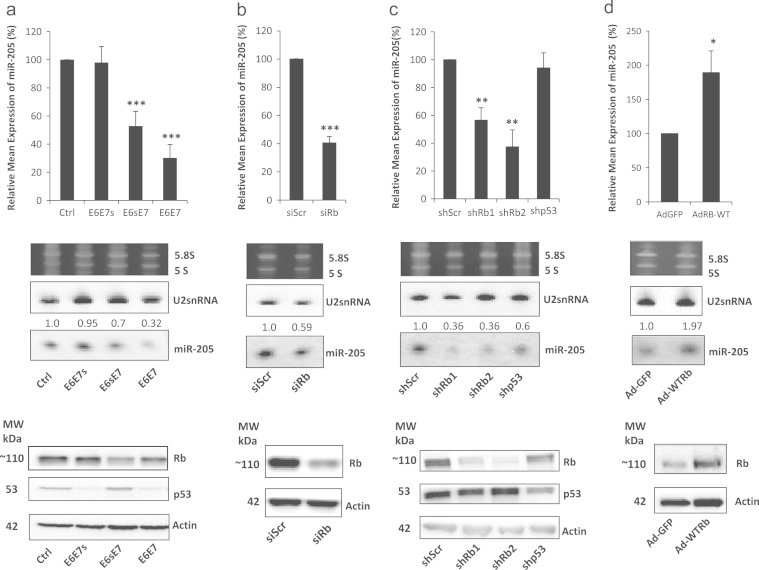
miR-205 is pRb dependent. (a) RQ-PCR and Northern blotting show that miR-205 expression is significantly decreased in HFKs expressing functional E7 alone (E6sE7) or with E6 (E6E7), compared to control (Ctrl) cells. However, in cells expressing E6 alone (E6E7s), no significant reduction is apparent. Western blotting for p53 and pRb demonstrates that both E6 and E7 are functional where expected. (b) RQ-PCR and Northern blotting show that transient knockdown of Rb using targeted siRNA (siRb) results in significant decrease of miR-205 compared to cells transfected with non-targeting scramble control (siScr). Western blotting for pRb confirms the knockdown. (c) RQ-PCR and Northern blotting show that stable knockdown of Rb using two targeted shRNA molecules (shRb1 and shRb2) results in significant decrease of miR-205 compared to cells expressing non-targeting scramble control (shScr). No significant decrease of miR-205 was observed in HFKs with stable knockdown of p53 (shp53). Western blotting for pRb and p53 confirms the respective knockdowns. (d) RQ-PCR and Northern blotting show that re-expressing Rb in shRb2 cells using an adenoviral construct (Ad-WT-Rb) results in a significant increase of miR-205 compared to shRB2 cells with control adenoviral construct (AdGFP). Western blotting for pRb confirms the re-expression. Numbers above miR-205 panels in Northern blots represent expression signal of miR-205 signal after normalization to U2snRNA expression. All images and blots are representative of three independent experiments performed on separate batches of HFKs. Data shown in graphs are mean±SE (Student *t*-test *p*-values: *p<0.05, **p<0.01, and ***p<0.001).

In our previous study of miR-203 expression in HFKs, we had demonstrated that miR-205 levels were not significantly altered by knockdown of p53 levels (McKenna et al., 2010). Since miR-205 levels were reduced in HFKs expressing E6 and E7 (Fig. 2a), we wished to examine if miR-205 expression was dependent upon pRb expression. Firstly, we examined if E7 expression, rather than E6, was responsible for determining miR-205 levels. Fig. 3a shows by RQ-PCR and northern blot that when E7 is expressed alone (E6sE7 – E6 with stop codon at 5′ end of E6) or with E6 (E6E7), miR-205 levels are significantly reduced. In contrast, when E6 alone is expressed (E6E7s – stop codon at 5′ end of E7), miR-205 levels are not altered, a finding that is in agreement with our previous observation that p53 levels do not affect miR-205. Next, we transiently knocked down pRb levels in HFKs and demonstrated that miR-205 levels were subsequently decreased as a result (Fig. 3b). To further validate this finding, we stably knocked down pRb in HFKs, using two separate targeting molecules, and again observed a similarly significant decrease in miR-205 levels (Fig. 3c). For comparison, we included HFKs with stable knockdown of p53, in which miR-205 levels were not altered, as we expected. Finally, we took one of the stable pRb knockdown cell lines and re-expressed wild-type pRb using an adenovirus construct. This rescued the pRb status of the cells and resulted in a restoration of the miR-205 expression (Fig. 3d). Together, this set of experiments provide strong evidence that miR-205 expression is dependent on pRb levels, and explains how E7 inhibition of pRb may result in decreased miR-205 levels. The mechanism causing this remains unclear; however, it would seem reasonable to speculate that E7 inactivation of pRb releases E2F transcription factors to induce target genes, which may include miRNAs. This has been shown to be the case for miR-15b in HPV-related anal carcinoma cells (Myklebust et al., 2011), but there are no reports of a similar effect on other miRNAs. However, in the case of miR-205, we see a reduction in expression, suggesting it is repressed rather than activated. A possible candidate for this repression is another miRNA, miR-184, which has been shown to antagonize miR-205 in corneal epithelial keratinocytes (Yu et al., 2008). Although no studies have investigated regulation of miR-184 activity, analysis of the promoter region upstream of miR-184 using rVISTA (Loots and Ovcharenko, 2004) reveals putative E2F and MYC-MAX binding sites (Supplementary Fig. 2a), so it is tempting to speculate that either E2F, or E2F-mediated induction of c-MYC expression, could result in increased miR-184 levels, which in turns represses miR-205 expression. In the case of miR-24, it is also feasible that inactivation of pRb also contributes to its up-regulation, since it is known to be up-regulated by c-MYC (Li et al., 2013) and has several putative E2F1 binding sites in the vicinity of the miR-23b–27b–24 cluster region (Supplementary Fig. 2b). Furthermore, a study by Mishra et al. (2009) suggested that miR-24 regulation was independent of p53 activity in cancer cells. However, some of our data (not shown) suggested that E6 and E7 separately resulted in increased miR-24 levels, but this data was not conclusive enough to allow us to draw similar conclusions for a relationship between miR-24 and p53 in HFKs. Ongoing work in our laboratory intends to explore this further.

**Fig. 4:**
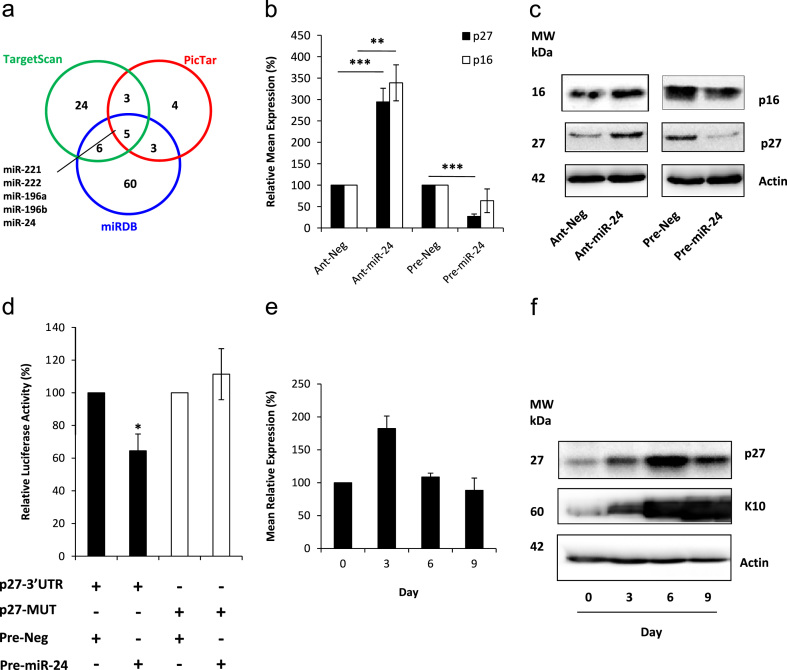
p27 is predicted to be a target of hsa-miR-24. (a) Venn diagram displaying numbers of miRNAs computationally predicted to target p27 (CDKN1B) by TargetScan (green) (http://www.targetscan.org/), PicTar (red) (http://pictar.mdc-berlin.de/) and miRDB (blue) (http://mirdb.org/). (b) RQ-PCR and (c) western blotting show that the inhibition of miR-24 expression in HFKs results in increased levels of p27, whilst over-expressing miR-24 in these cells results in significantly decreased levels of p27. p16, a known target of miR-24, is shown for comparison. (d) Luciferase activity reporter confirms miR-24 targeting of p27 in HFKs. A reporter construct containing the wild-type p27 3′UTR region (p27-3′UTR, black bars) shows significant reduction of luciferase activity when co-transfected with precursor miR-24 (pre-miR-24) relative to cells co-transfected with non-targeting control (pre-neg). In cells transfected with a construct containing mutated residues in the miR-24 binding site of p27 3′UTR (p27-MUT, white bars), no reduction in luciferase activity is observed. (e) RQ-PCR and (f) western blotting for p27 in RNA and protein samples harvested from organotypic rafts at different time points shows p27 levels initially rise, before decreasing again over the differentiation time. Data shown in figures (b–d) is representative of three independent experiments performed on separate batches of HFKs. Figures (e) and (f) are representative of two independent experiments performed on separate batches of HFKs. Data in graphs is mean±SE (Student *t*-test *p*-values: ^⁎^*p*<0.05, ^⁎⁎^*p*<0.01, and ^⁎⁎⁎^*p*<0.001).

We were also interested in the potential targets of miR-24 within the cell. The observation that p27 levels were increased when miR-24 was knocked down prompted us to investigate whether it was a potential target of miR-24. Using three miRNA target prediction algorithm programs, we found that miR-24 was consistently predicted to target p27 (Fig. 4a and Supplementary Fig. 3). To test this relationship *in vitro*, we again knocked down miR-24 levels in HFKs and demonstrated by RQ-PCR (Fig. 4b) and western blotting (Fig. 4c) that p27 levels were significantly increased as a result. In the reverse experiment, we over-expressed miR-24 levels in HFKs and demonstrated that p27 levels were significantly reduced (Fig. 4b and c). As a control, we quantified levels of p16, a known target of miR-24 (Lal et al., 2008). The fact that altering miR-24 levels results in a similar pattern of expression for p27 suggested that it may also be a target and we proceeded to confirm this with a luciferase reporter assay (Fig. 4d). The luciferase activity of a reporter construct containing the wild-type p27 3′UTR region (p27-3′UTR) showed significant reduction when miR-24 was over-expressed in the same cells, indicating that miR-24 was binding to the target region in the 3′UTR of p27 mRNA (as shown in Supplementary Fig. 3). However, when miR-24 was over-expressed with a reporter construct which had mutated residues in the miR-24 binding site of p27 3′UTR (p27-MUT), no reduction in luciferase activity was observed. This is strong evidence that miR-24 does indeed regulate p27 levels in keratinocytes. Our observations are corroborated by a recent study by Giglio et al. (2013), published while this manuscript was being prepared, which also demonstrates that miR-24 targets p27 in keratinocytes. Since we had confirmed this interaction we were then interested to see if miR-24 levels inversely correlated with p27 levels during differentiation, so we quantified p27 by RQ-PCR (Fig. 4e) and western blotting (Fig. 4f) in samples from our organotypic raft model. However, we did not observe an exact correlation since p27 protein levels increase until Day 6, before decreasing, where we might have expected the increasing levels of miR-24 to result in decreased p27 levels from Day 0 to Day 9. However, we had noted a similar delayed response during differentiation between miR-203 and its target p63 (McKenna et al., 2010), so we suggest that miR-24 may still contribute to the control of p27 levels during HFK differentiation, although other mechanisms are likely to also play a role and presumably override the regulation of p27 by miR-24 during the early period of differentiation. Indeed, we are inclined to speculate that miR-24 may play differing functions in cycling and differentiating cells. In cycling cells, the increased miR-24 levels appear to be associated with the increased proliferation which is exhibited by cycling cells expressing E6 and E7 compared to control cells. Association of miR-24 with proliferation is an observation which is supported by our findings in Fig. 2(h) and it also agrees with observations by others, who have shown that miR-24 promotes cell proliferation in different settings (Lin et al., 2010, Giglio et al., 2013).

However, as we noted in our introduction, miR-24 can play contrasting roles depending on the setting and there is evidence from other studies that miR-24 can also inhibit cell proliferation (Amelio et al., 2012, Mishra et al., 2009). This is apparently the case in the differentiation of normal HFKs, where an increase in miR-24 correlates with decreased proliferation, whilst the lack of miR-24 induction noted in cells expressing E6 and E7 associates with increased proliferation. It is possible that a switch in miR-24 function therefore occurs during HFK differentiation, whereby proliferation is inhibited instead of promoted. Presumably, this might be due to the effect of miR-24 on other targets which would override the proliferative effect of miR-24 noted in cycling cells. In this study, we have focused on p27 and p16 as targets of miR-24, but we fully expect other (known and, as yet, unknown) targets of miR-24 to be also affected, any of which might also contribute to control of differentiation. Nor can we exclude the possibility that other miRNAs play a role, including miR-23b and 27b from the same cluster as miR-24, and also other miRNAs which target p27. These possible contributing factors may go some way to explaining why we find p27 levels do not exactly correlate with miR-24 levels during differentiation.

## Conclusions

In summary, we have provided further data supporting the evidence that that miR-24 and miR-205 play important roles in keratinocytes. We have also shown that the levels of both miRNAs can be altered by expression of HPV onco-proteins in HFKs. The expression of miR-205 is dependent upon pRb levels, which means it is susceptible to alteration by E7 activity. Meanwhile, miR-24 is apparently up-regulated by E6 and E7 expression and may promote cell proliferation by targeting the cell cycle inhibitor p27. These observations provide new evidence as to how HPV infection can lead to deregulation of proliferation and differentiation in keratinocytes during the development of cervical cancer.

## Materials and methods

### Cell culture, infections and transfections

Primary human foreskin keratinocytes (HFKs) were isolated from neonatal foreskin, cultured in low calcium and transduced with retrovirus produced in ΦNYX-GP packaging cell-line (ATCC) as previously described (Incassati et al., 2006). Mutagenesis of E6 or E7 was performed as previously described to generate a stop codon at the 16th amino acid in E7 gene of HPV-16 (E6E7s) (Guess and McCance, 2005), a stop codon at the 15th amino acid in E6 gene of HPV-16 (E6sE7) (Guess and McCance, 2005). For stable knockdown of pRb and p53, pSuper-retro constructs expressing short hairpin RNAs (shRNA) against no known annotated gene (shScr), 2 regions of Rb 3′UTR (shRb1 and shRb2) and p53 3′UTR (shp53) were cloned as described previously (Incassati et al., 2006). Recombinant adenoviruses were cloned using ViraPower Adenoviral Gateway Expression Kit (Invitrogen) as previously described (Pickard et al., 2010). For siRNA transfection, control and RB1 siRNA molecules were purchased from Ambion (Warrington, UK). Transfection was performed at a final concentration of 200 nM using FuGene HD for up to 48 h.

Differentiation of HFK cell lines in organotypic raft cultures was carried out as previously described for transduced lines (McCance et al., 1988). For calcium-induced differentiation, confluent monolayers of HFKs were induced to differentiate by withdrawal of growth factors and addition of 1.5 mM CaCl_2_. Transfection of HFKs with anti-miR-24, pre-miR-24, anti-miR-205, pre-miR-205 and negative controls (all Ambion) was performed using FuGene HD (Roche, Mannheim, Germany) following manufacturer's protocols. Cells were transfected for 48 h with a final concentration of 50 nM. In order to label DNA synthesizing cells, cells in culture were pulsed for 20 mins with 10 μm BrdU prior to fixation.

### Immunofluorescent analysis

BrdU pulsed cells on coverslips were fixed for 10 mins with 4% paraformaldehyde, washed 3× with PBS, submitted to antigen retrieval and stained with anti-BrdU (BD Biosciences, Oxford, UK), A minimum of 10 fields of view and >500 cells were counted for each slide using a Leica AF6000 inverted fluorescence microscope and Leica AF imaging software. BrdU graphs represent mean±SE of three independent experiments, expressed relative to number of BrdU incorporating cells in control experiments.

### Luciferase reporter assay

Luciferase reporter constructs based on the pMirTarget firefly luciferase plasmid were purchased from OriGene Technologies (Rockville, MD). One construct contained the wild-type p27 3′UTR region with the miR-24 binding site intact (p27-3′UTR). A matched control construct contained 2 mutated bases in the miR-24 binding site (cataCTGAGCCAagtat changed to cataCTGTACCAagtat) (p27-MUT). HFKs were seeded at a concentration of 100,000 cells/well in 12 well plates and transfected with 500 ng of either p27-3′UTR plasmid of p27-MUT, together with either pre-miR-24 or non-targeting negative control (pre-neg) at a concentration of 50 nM. FuGene HD was used for the transfection and 50 ng Renilla luciferase vector was included in each well to control for transfection efficiency. After 48 h, cells were lysed in lysis buffer (Promega, Southampton, UK) and luciferase activity measured using the Dual-Glo^®^ Luciferase Assay Kit (Promega) on a FluoStar Omega plate reader (BMG LabTech, Aylesbury, UK). Transfections were carried out in triplicate, measurements within experiments were performed in duplicate, and firefly luciferase readings were normalized against renilla luciferase readings before analysis.

### Western blot analysis

Protein lysates were electrophoresed and equal loading assessed by Ponceau Red staining following transfer to nitrocellulose membrane. Primary antibodies used for blotting were anti-pRb, anti-p16, anti-p21Cip1 (all BD Biosciences), anti-p53(DO-1), anti-p27 (both Santa Cruz), anti-K1, anti-K10 (both Covance, Cambridge, UK), Cyclin D1, AKT, pAKT(ser473) (all Cell Signaling, Hertfordshire, UK) and anti-β-actin (Sigma, Poole, UK) as loading control. Secondary antibodies were goat anti-mouse- and anti-rabbit-HRP (Santa Cruz). Luminescence was revealed by incubation with either (Perkin-Elmer) or (Pierce) and signal detected on an Alpha Innotech FluorChem^™^ SP imaging system.

### RT-PCR analysis

RNA extraction was carried out with High Pure RNA isolation kit (Roche) according to manufacturer's instructions. 1 μg RNA was treated with RQ1 RNAse free DNAse (Promega) prior to first strand cDNA synthesis using random primers with transcriptor high-fidelity cDNA synthesis kit (Roche) according to manufacturer's instructions. For real-time PCR, amplification of PCR products was quantified using FastStart SYBR Green Master (Roche) according to manufacturers instruction and fluorescence monitored on a DNA Engine^®^ Peltier Thermal Cycler (Bio-Rad) equipped with a Chromo4 Real-Time PCR Detection System (Bio-Rad) and melting curve analysis also performed. The cycles of 95 °C – 15 s, 58 °C – 15 s, 60 °C – 60 s, using primer sets for p27 (Forward 5′–TTTGACTTGCATGAAGAGAAGC-3′; Reverse 5′–AGCTGTCTCTGAAAGGGACATT–3′), p16 (forward 5′–GTGGACCTGGCTGAGGAG-3′; reverse 5′–CTTTCAATCGGGGATGTCTG-3′) and RPLPO (5′–ATCAACGGGTACAAACGAGTC–3′; reverse 5′–CAGATGGATCAGCCAAGAAGG–3′). Expression levels were assessed in triplicate, normalized to RPLPO levels and graphs represent the combined results of three independent biological replicates.

### microRNA real-time PCR

Real-time quantitative PCR (RQ-PCR) of miRNAs was performed using the miRCURY LNA^™^ microRNA PCR system (Exiqon, Vedbaek, Denmark). 10 ng template RNA was used in each first strand cDNA synthesis reaction. PCR was performed over 40 amplification cycles and fluorescence monitored as described above. Analysis was performed using the Opticon Real-Time PCR Detection System (Bio-Rad). For all RQ-PCR analysis, normalization was against U6snRNA and error bars represent±SE from three independent experiments.

### Northern blotting for microRNAs

Total RNA was extracted from cells using Trizol (Invitrogen, Paisley, UK) and quantified by spectophotometric analysis. Northern blotting was performed by resolving 10 µg total RNA on denaturing polyacrylamide TBE-Urea 15% gels (Invitrogen), transferring onto BrightStar-Plus positively charged nylon membrane (Ambion), followed by UV cross-linking. The membrane was hybridized overnight at 42 °C with DIG-labelled LNA probe specific for miR-24 or miR-205 (0.1 nM) (Exiqon) or DIG-labelled antisense probe to U2snRNA (GGGTGCACCGTTCCTGGAGGTAC) (100 ng/ml). Following post-hybridization washing, signal detection was performed using the DIG luminescent Detection Kit (Roche). Signal was detected on an Alpha Innotech FluorChem^™^ SP imaging system.

### Statistics

Experiments were carried out at least three times, unless otherwise indicated. Two-tailed Student's *t*-test was used to calculate *p*-values, with thresholds of ^⁎⁎⁎^*p*<0.001, ^⁎⁎^*p*<0.01, and ^⁎^*p*<0.05.

## Footnotes

Appendix A Supplementary data associated with this article can be found in the online version at doi:10.1016/j.virol.2013.10.014.

## References

[bib1] Amelio I., Lena A.M., Viticchiè G., Shalom-Feuerstein R., Terrinoni A., Dinsdale D., Russo G., Fortunato C., Bonanno E., Spagnoli L.G., Aberdam D., Knight R.A., Candi E., Melino G. (2012). miR-24 triggers epidermal differentiation by controlling actin adhesion and cell migration. J. Cell Biol..

[bib2] Andl T., Murchison E.P., Liu F., Zhang Y., Yunta-Gonzalez M., Tobias J.W., Andl C.D., Seykora J.T., Hannon G.J., Millar S.E. (2006). The miRNA-processing enzyme dicer is essential for the morphogenesis and maintenance of hair follicles. Curr. Biol..

[bib3] Bauersachs J., Thum T. (2007). MicroRNAs in the broken heart. Eur. J. Clin. Invest..

[bib4] Cowland J.B., Hother C., Grønbaek K. (2007). MicroRNAs and cancer. Acta Pathol. Microbiol. Immunol. Scand..

[bib5] Erson A.E., Petty E.M. (2008). MicroRNAs in development and disease. Clin. Genet..

[bib6] Giglio S., Cirombella R., Amodeo R., Portaro L., Lavra L., Vecchione A.J. (2013). MicroRNA miR-24 promotes cell proliferation by targeting the CDKs inhibitors p27(Kip1) and p16(INK4a). Cell Physiol..

[bib7] Guess J.C., McCance D.J. (2005). Decreased migration of Langerhans precursor-like cells in response to human keratinocytes expressing human papillomavirus type 16 E6/E7 is related to reduced macrophage inflammatory protein-3alpha production. J. Virol..

[bib8] Incassati A., Patel D., McCance D.J. (2006). Induction of tetraploidy through loss of p53 and upregulation of Plk1 by human papillomavirus type-16 E6. Oncogene.

[bib9] Kim J.S., Yu S.K., Lee M.H., Park M.G., Park E., Kim S.G., Lee S.Y., Kim C.S., Kim H.J., Chun H.S., Chun S.W., Kim do K. (2013). MicroRNA-205 directly regulates the tumor suppressor, interleukin-24, in human KB oral cancer cells. Mol. Cells..

[bib10] Lal A., Kim H.H., Abdelmohsen K., Kuwano Y., Pullmann R., Srikantan S., Subrahmanyam R., Martindale J.L., Yang X., Ahmed F., Navarro F., Dykxhoorn D., Lieberman J., Gorospe M. (2008). p16(INK4a) translation suppressed by miR-24. PLoS One.

[bib11] Latronico M.V., Catalucci D., Condorelli G. (2007). Emerging role of microRNAs in cardiovascular biology. Circ. Res..

[bib12] Li X., Liu X., Xu W., Zhou P., Gao P., Jiang S., Lobie P.E., Zhu T. (2013). c-MYC-regulated miR-23a/24-2/27a cluster promotes mammary carcinoma cell invasion and hepatic metastasis by targeting sprouty2. J. Biol. Chem..

[bib13] Lin S.C., Liu C.J., Lin J.A., Chiang W.F., Hung P.S., Chang K.W. (2010). miR-24 up-regulation in oral carcinoma: positive association from clinical and in vitro analysis. Oral Oncol..

[bib14] Loots G., Ovcharenko I. (2004). rVista 2.0: evolutionary analysis of transcription factor binding sites. Nucl. Acids Res..

[bib15] McCance D.J., Kopan R., Fuchs E., Laimins L.A. (1988). Human papillomavirus type 16 alters human epithelial cell differentiation in vitro. Proc. Natl. Acad. Sci. USA.

[bib16] McKenna D.J., McDade S.S., Patel D., McCance D.J. (2010). MicroRNA 203 expression in keratinocytes is dependent on regulation of p53 levels by E6. J. Virol..

[bib17] Mishra P.J., Song B., Mishra P.J., Wang Y., Humeniuk R., Banerjee D., Merlino G., Ju J., Bertino J.R. (2009). MiR-24 tumor suppressor activity is regulated independent of p53 and through a target site polymorphism. PLoS One.

[bib18] Myklebust M.P., Bruland O., Fluge Ø., Skarstein A., Balteskard L., Dahl O. (2011). MicroRNA-15b is induced with E2F-controlled genes in HPV-related cancer. Br. J. Cancer..

[bib19] Nissan X., Denis J.A., Saidani M., Lemaitre G., Peschanski M., Baldeschi C. (2011). miR-203 modulates epithelial differentiation of human embryonic stem cells towards epidermal stratification. Dev. Biol..

[bib20] Papadimitriou E., Vasilaki E., Vorvis C., Iliopoulos D., Moustakas A., Kardassis D., Stournaras C. (2012). Differential regulation of the two RhoA-specific GEF isoforms Net1/Net1A by TGF-β and miR-24: role in epithelial-to-mesenchymal transition. Oncogene.

[bib21] Pickard A., Wong P.P., McCance D.J. (2010). Acetylation of Rb by PCAF is required for nuclear localization and keratinocyte differentiation. J. Cell Sci..

[bib22] Qin A.Y., Zhang X.W., Liu L., Yu J.P., Li H., Wang S.Z., Ren X.B., Cao S. (2013). MiR-205 in cancer: an angel or a devil?. Eur. J. Cell Biol..

[bib23] Sullivan C.S., Ganem D. (2005). MicroRNAs and viral infection. Mol. Cell.

[bib24] Visone R., Croce C.M. (2009). MiRNAs and cancer. Am. J. Pathol..

[bib25] Yi R., O′Carroll D., Pasolli H.A., Zhang Z., Dietrich F.S., Tarakhovsky A., Fuchs E. (2006). Morphogenesis in skin is governed by discrete sets of differentially expressed microRNAs. Nat. Genet..

[bib26] Yu J., Peng H., Ruan Q., Fatima A., Getsios S., Lavker R.M. (2010). MicroRNA-205 promotes keratinocyte migration via the lipid phosphatase SHIP2. FASEB J..

[bib27] Yu J., Ryan D.G., Getsios S., Oliveira-Fernandes M., Fatima A., Lavker R.M. (2008). MicroRNA-184 antagonizes microRNA-205 to maintain SHIP2 levels in epithelia. Proc. Natl. Acad. Sci. USA.

[bib28] Zheng Z.M., Wang X. (2011). Regulation of cellular miRNA expression by human papillomaviruses. Biochim. Biophys. Acta.

