# Supplementary material for: miR-24 and miR-205 expression is dependent on HPV onco-protein expression in keratinocytes
Source: Virology. 2014 Jan 5;448:210–6. doi: 10.1016/j.virol.2013.10.014 (PMC3865362; doi:10.1016/j.virol.2013.10.014)

Supplemental Table 1. Differential expression of selected miRNAs due to differentiation in HFKs.

Sample ID	Average Mean Fluorescence Intensity	Mean Fold change due to differentiation
miR-205	1608.5	1.69
miR-24	451.5	1.68
miR-31	361.75	1.25
miR-211	342.75	1.05
miR-221	210.25	1.25
miR-23a	207.5	1.88
miR-29a	197.5	1.2
miR-125b	172.75	1.52
miR-23b	159.5	1.75
miR-181a	154.75	0.84
miR-27a	142	2.25
miR-92	138.5	1.18
miR-200c	137.75	1.14
miR-412	137.25	1.03
let-7b	133.25	1.64
miR-215	127	1.96
miR-325	121.5	1.14
let-7a	117.5	1.04
miR-22	116	1.69
miR-181b	111.75	0.79
miR-320	110.5	1.25
miR-30a-5p	110	1.07
miR-203	108.5	2.53

Fig 1 Supplemental

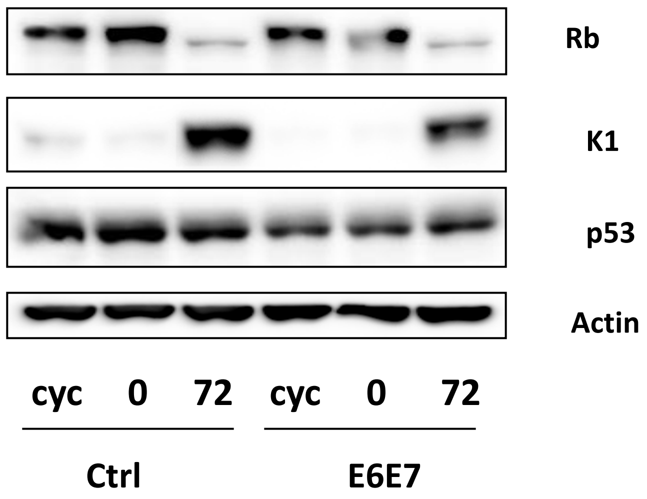

Fig 2 Supplemental

(a)

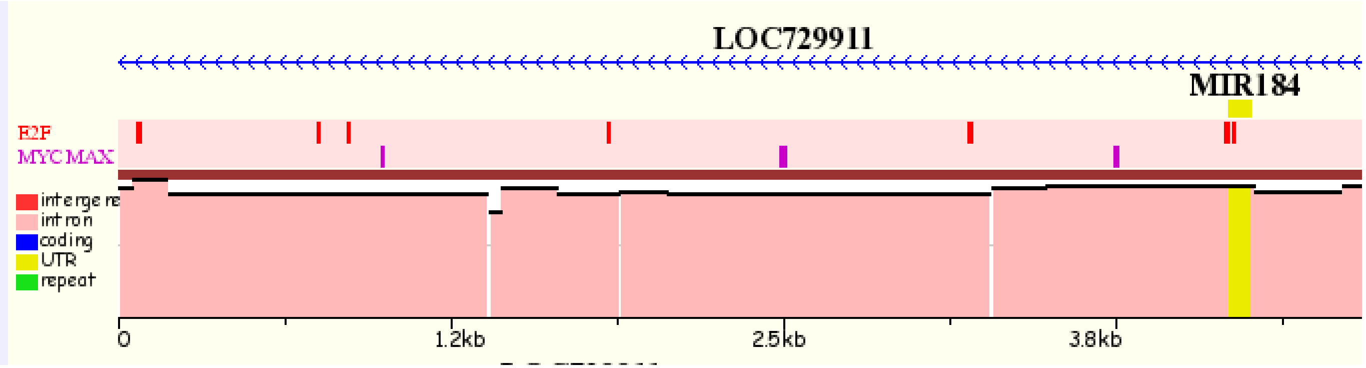

(b)

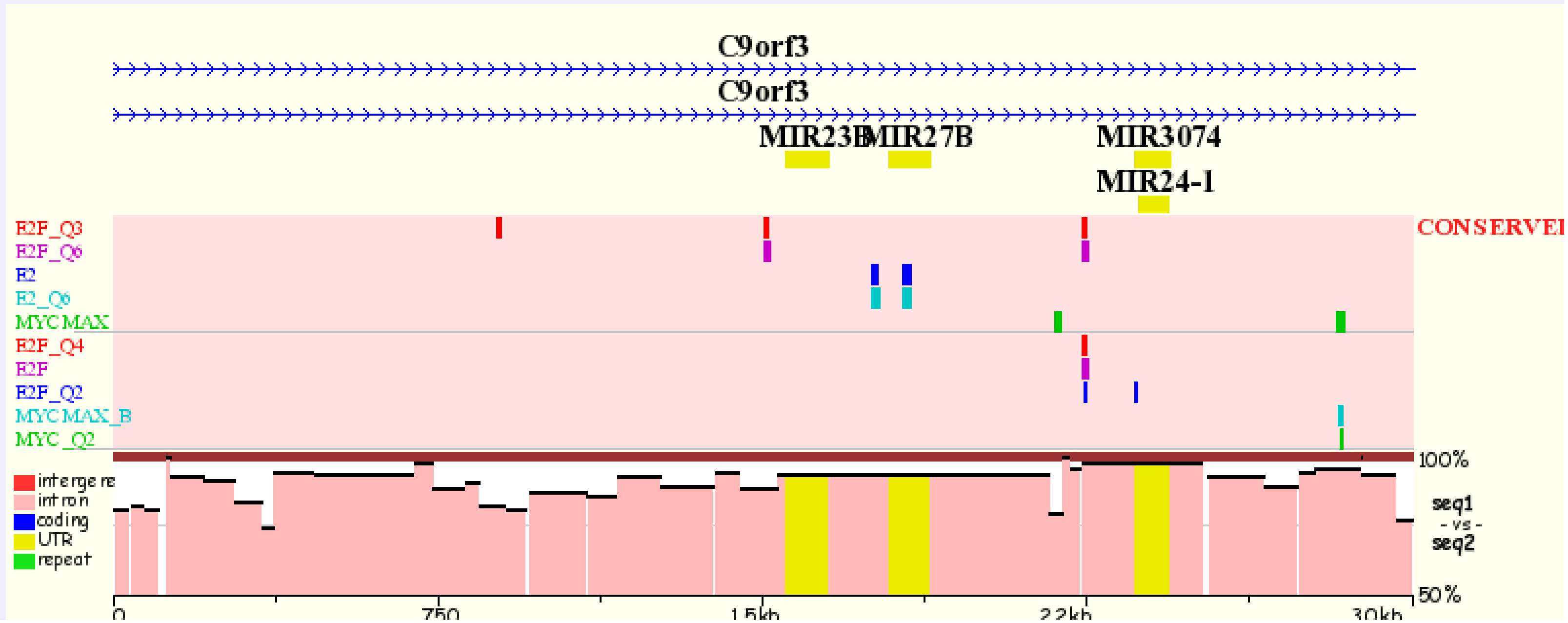

Fig 3 Supplemental

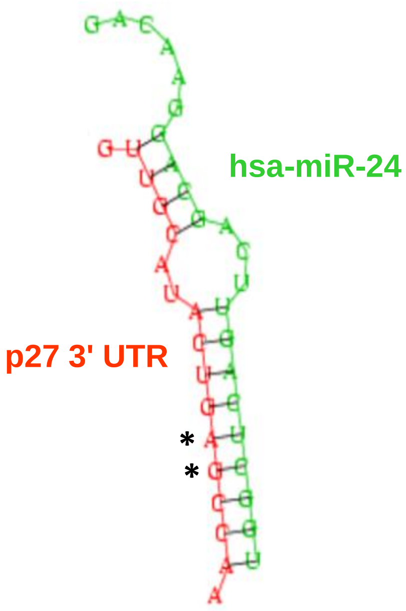

Supplement: Supplementary file 1 — Supplementary Material [file mmc1.pdf]
